# The ascending aortic image quality and the whole aortic radiation dose of high-pitch dual-source CT angiography

**DOI:** 10.1186/1749-8090-8-228

**Published:** 2013-12-12

**Authors:** Ying Liu, Jian Xu, Jian Li, Jing Ren, Hongtao Liu, Junqing Xu, Mengqi Wei, Yuewen Hao, Minwen Zheng

**Affiliations:** 1 Department of Radiology, Xijing Hospital, Fourth Military Medical University, 169 West Changle Road, Xi’an 710032, China; 2 Siemens China Ltd., Shang Hai, China

**Keywords:** Aorta, Radiation dose, Image quality, Dual-source CT

## Abstract

**Background:**

Aortic dissection is a lift-threatening medical emergency associated with high rates of morbidity and mortality. The incidence rate of aortic dissection is estimated at 5 to 30 per 1 million people per year. The prompt and correct diagnosis of aortic dissection is critical. This study was to compare the ascending aortic image quality and the whole aortic radiation dose of high-pitch dual-source CT angiography and conventional dual-source CT angiography.

**Methods:**

A total of 110 consecutive patients with suspected aortic dissection and other aortic disorders were randomly divided into two groups. Group A underwent traditional scan mode and Group B underwent high-pitch dual-source CT scan mode. The image quality and radiation dose of two groups were compared.

**Results:**

Close interobserver agreement was found for image quality scores (κ = 0.87). The image quality of ascending aorta was significantly better in the high-pitch group than in the conventional group (2.78 ± 0.46 vs 1.57 ± 0.43, *P* < 0.001). There was no significant difference of the CT attenuation values, the aortic image noise and SNR between two groups. The mean radiation dose of high-pitch group was also significantly lower than that of conventional group (2.7 ± 0.6 mSv vs. 3.9 ± 0.9 mSv, *P* < 0.001).

**Conclusions:**

High-pitch dual-source CT angiography of the whole aorta can provide motion-artifact-free imaging of the ascending aorta at a low radiation dose compared to conventional protocol.

## Background

With the rapid development of spiral technology and scan speed, the multi-slice spiral CT angiography (CTA) is more widely used for aortic emergencies, and has become the preferred method in aortic diseases [1-8]. However, the images of intimal tear location and intimal flap in type I and type II aortic dissection involving the ascending aorta often cannot satisfy clinical diagnosis because of the proximal segment of the pulsation artifact [9,10]. In addition, images of the ascending aorta are blurred using 64-slice CT or dual-source CT (DSCT) with earlier technology due to insufficient temporal resolution. On the other hand, due to the long scanning range and the patients’ preoperative assessment and postoperative follow-up for many times, the X-ray radiation hazards are also of concern [11,12]. How to reduce aortic CTA scanning radiation dose and how to improve image quality of ascending aorta has important meaning for clinical practice.

DSCT is characterized by two X-ray tubes and two corresponding detectors offset by approximately 90°, whose time resolution increases two times compared to single source CT [13,14]. The second generation DSCT system is equipped with a high-pitch data acquisition mode with pitch values of up to 3.4 by filling the gaps with the data acquired with the second detector system [15,16]. This high pitch acquisition mode is fast, reducing the scan time for the entire aorta to only about 2 seconds [17,18] and allowing for low radiation dose. Also, this CT system has improved gantry rotation time up to 0.28 s, which leads to a high temporal resolution of 75 ms for each acquired slice [19]. Because of this fast volume coverage and high temporal resolution, it might be feasible to acquire diagnostic image quality of ascending aorta even without the application of ECG-gating and to reduce radiation dose at the same time. However, this has not been investigated so far. Also, the CTA scan with ECG-gated high-pitch mode always need the heart rate with limit of 70 beats per minute, however, these critically ill patients with aortic dissection always have high heart rate and are not suitable for waiting to control the heart rate. This study was to evaluate the aortic radiation dose and image quality in the ascending aorta using high-pitch mode without ECG-trigger, compared with conventional aortic scan mode of DSCT, aiming to reduce radiation dose and improve the image quality of the ascending aorta at the same time.

## Methods

### Patients

One hundred ten consecutively registered patients (74 male, 36 female; age 57.7 ± 11.5 years, range 27~83 years) suspected aortic dissection and other aortic disorders were included during the study time frame (June 2011 through July 2012). They all presented with a sudden severe chest pain with a tearing or ripping sensation. The patients were randomly divided into Group A (n = 55) and Group B (n = 55), and underwent the DSCT aortic CTA examination. General exclusion criteria for contrast-enhanced CTA were nephropathy (defined as a serum creatinine level >150 mol/l) or a known hypersensitivity to iodinecontaining contrast agents. The study protocol was approved by the institutional review board of Xijing Hospital, Fourth Military Medical University, and written informed consent was obtained from all subjects. Also, the study was conducted in accordance with the Declaration of Helsinki.

### CT data acquisition

All examinations were performed with a second-generation DSCT system (Somatom Definition Flash, Siemens Healthcare, Germany). Thoracoabdominal scout view and contrast scan were performed from thoracic inlet to the pubic symphysis encompassing the chest. Each patient received an injection of 65 mL of iopromide (Ultravist 370, 370 mg I/mL, Bayer Schering Pharma) at a flow rate of 5 mL/s followed by 30 mL saline solution. A craniocaudal scan direction was chosen, and all CT imaging data were acquired with a breath hold in deep inspiration to eliminate respiratory motion artifacts. In Group A, conventional single tube scan was used with gantry rotation time 0.5 s, pitch 1.2 and a tube current time product of 110 reference mAs/rotation. Contrast administration was controlled with bolus tracking. The region of interest was placed in the aortic root. In Group B, high-pitch CTA scan was used with gantry rotation time 0.28 s, pitch 3.2 and quality reference mAs of 110. Contrast administration also was controlled with bolus tracking. The region of interest was placed in the common iliac artery bifurcation. The automatic trigger scan started 6 seconds after the attenuation threshold of 100 HU in both groups. All the other scanning parameters in both groups were as follows: detector collimation 2 × 64 × 0.6 mm, slice acquisition 2 × 128 × 0.6 mm by means of a z-flying focal spot, gantry rotation time 280 ms, tube voltage 100 kV, matrix 512 × 512, and FOV 200~320 mm. Automatic exposure control, CareDose4D, was used in all exams to adjust scanner output according to patient size.

### Postprocessing and image reconstruction

All the original scan data were transmitted to the Siemens workstation (SyngoMMWP VE40A, Siemens Medical Solutions) for image postprocessing. Images were reconstructed using multiplanar reconstruction (MPR) with a slice thickness of 1.0 mm and an increment of 0.8 mm with a medium smooth tissue convolution kernel (B26f).

### Evaluation of image quality

#### Subjective evaluation method

Image quality of ascending aorta was assigned separately by two independent experienced attending radiologists. The reviewers gave the examinations an overall grade according to the following scale: 3, excellent quality without limitation; 2, moderate quality with mild blurring; or 1, significant blurring or doubled appearance of structures [20]. Motion artifacts were assessed for the ascending aorta.

#### Objective evaluation methods

The aortic CT enhancement values, the aortic image noise and background noise were measured in the region of interest with 30 mm^2^ in the aortic arch and iliac artery bifurcation, respectively. The signal to noise ratio (SNR) was calculated according to the following equation: SNR = SI_aorta_ /SD, where SI_aorta_ is the aortic CT enhancement values and SD is the background noise.

### Evaluation of radiation dose

The volume CT dose index (CTDIvol) and the dose length product (DLP) were recorded, which were measured automaticlly by CT scanner. According to the DLP was used to calculate the effective dose (ED). ED = k × DLP. k coefficient was 0.015 according to the trunk value [21].

### Statistical analysis

All data analyses were performed with statistical software (SPSS 17.0, SPSS). Quantitative data were expressed as mean ± SD and categorical variables were expressed as frequencies or percentages. T test was used to determine whether there was a significant difference between radiation dose and image noise of two groups. Interobserver agreement on the image quality was evaluated with Cohen’s Kappa statistics. A κ value greater than 0.81 was interpreted as excellent interobserver agreement, values of 0.61-0.80 were interpreted as good, values of 0.41-0.60 as moderate, values of 0.21-0.40 as fair and values less than 0.20 as poor agreement. Pairwise comparison between groups was performed by using the Mann-Whitney paired-sample test. A value of *P* < 0.05 was considered statistically significant.

## Results

### Patients and image findings

The final diagnosis of 110 patients was summarized in Table 1. Ninety three patients (59 male, 34 female; mean age: 56.2 ± 11.1 years; range 27 to 81 years) were diagnosed aortic dissection by aortic CTA in this study, including 45 cases in Group A and 48 cases in Group B. They were all divided into 3 types according to DeBakey classification. The intimal tear of Type I and II occured in the the ascending aorta near the sinotubular junction, which coincided with intraoperative findings. In Group A, there were 13 cases of type I aortic dissection, 5 cases of type II and 27 cases of type III. True and false lumen and intimal flap were clearly displayed and the display rate was 100% (45/45) compared to surgical findings. Also, the display rate of aortic dissection tear was 93.3% (42/45). Due to the pulsation artifacts of ascending aortic root, the intimal tear of three cases could not be clearly shown or measured. Moreover, the coronary arteries could not be clearly shown. In Group B, there were 16 cases of type I, 4 cases of type II and 28 cases of type III. The display rate of true and false lumen and intimal flap was also 100% (48/48). The display rate of aortic dissection tear was 97.9% (47/48). Only one intimal tear could not be clearly shown because of the pulsation artifacts of ascending aortic root. Only 18 openings of coronary arteries could be clearly displayed in Group B, in which there were 5 artery dissection of left main trunk and 1 coronary artery dissection of right coronary artery.

**Table 1:** 110 patients diagnosis of traditional scan mode (Group A) and high-pitch scan mode (Group B)

	**AD**			**True aneurysm**	**Pseudoaneurysm**	**Normal**	**Total**
	**I**	**II**	**III**				
Group A	13	5	27	5	1	4	55
Group B	16	4	28	4	0	3	55

*AD*, aortic dissection.

### Scanning parameters and radiation dose

The basic scanning parameters and radiation dose of two groups were shown in Table 2. With the same basic scanning parameters as Group A, the patients in Group B received less radiation dose and shorter scanning time, which were suitable for emergency and patients who were hard to cooperate with.

**Table 2:** Scanning parameters and radiation dose of traditional scan mode (Group A) and high-pitch scan mode (Group B)

	**Group A**	**Group B**	***P *** **value**
Body mass index (kg/m^2^)	24.3 ± 3.4	24.9 ± 2.7	0.95
Scan length (mm)	607.2 ± 37.9	610.1 ± 40.1	0.71
Scan time (s)	7.8 ± 0.9	1.6 ± 0.1	< 0.001
CTDIvol (mGy)	4.0 ± 0.8	2.7 ± 0.5	< 0.001
ED (mSv)	3.9 ± 0.9	2.7 ± 0.6	< 0.001

*CTDIvol*, volume CT dose index; *ED*, effective dose.

### Interobserver agreement and image quality

**Figure 1:**
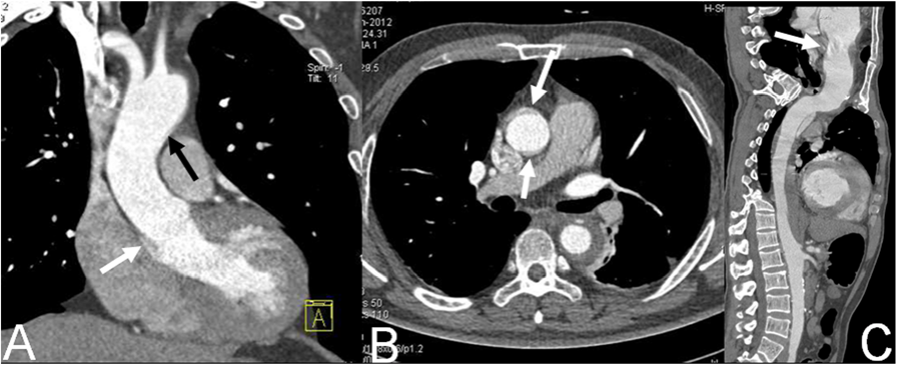
**Dual-source CT imaging with traditional mode.** Coronal MPR image **(A)** shows pulsation artifact of the aortic wall of distal ascending aorta (black arrow) and aortic sinus (white arrow). Axial original image **(B)** and CPR image **(C)** show the pulsation artifact of the ascending aortic wall (white arrows).

**Figure 2:**
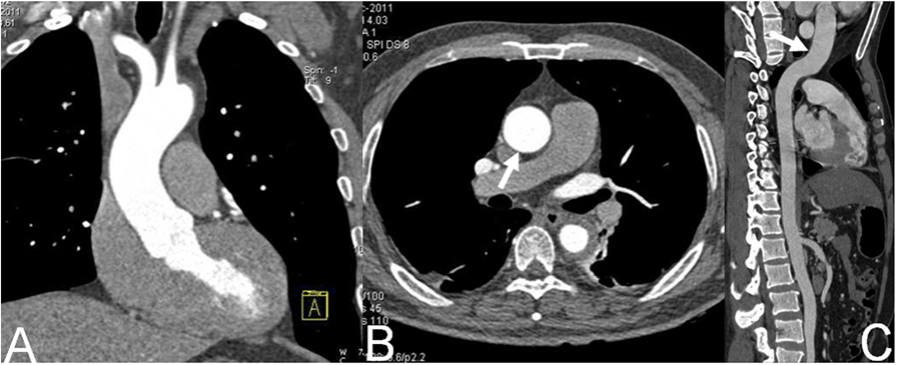
**Dual-source CT imaging with high-pitch mode.** Coronal MPR image (**A**, white arrow), axial original image (**B**, white arrow) and CPR image **(C)** show the wall of the entire ascending aorta is smooth without pulsation artifact.

All interobserver agreements for image quality assessment were good (κ = 0.87). No systematic difference between the two observers’ results was found during data analysis. Pulsation artifacts of ascending aorta were significantly reduced in Group B, and there was significant difference of image quality score between two groups (1.57 ± 0.43 vs 2.78 ± 0.46, *P* < 0.001, Figure 1 and Figure 2).

### Image noise

The aortic CT enhancement values and the aortic image noise were shown in Table 3. There was no significant difference of the CT attenuation values, the aortic image noise and SNR between two groups.

**Table 3:** The aortic CT enhancement values and the aortic image noise of traditional scan mode (Group A) and high-pitch scan mode (Group B)

	**Group A**	**Group B**	***P *** **value**
CT enhancement values of aortic arch (HU)	476.0 ± 93.3	448.5 ± 87.4	0.96
Image noise of aortic arch	22.6 ± 9.2	20.4 ± 5.3	0.12
CT enhancement values of iliac artery bifurcation (HU)	447.6 ± 93.2	432.6 ± 83.6	0.42
Image noise of iliac artery bifurcation	31.8 ± 12.2	28.3 ± 7.6	0.10
SNR	27.8 ± 8.7	25.9 ± 9.3	0.25

*SNR*, signal to noise ratio.

## Discussion

Aortic dissection is the most frequently diagnosed lethal condition of the aorta, especially in the acute setting [22-25]. Prompt and correct diagnosis of aortic dissection is critical for rapid and timely treatment and prognosis, which can decrease mortality and increase the survival rate. Currently, multislice CT imaging is the method of choice for diagnosis of aortic dissection due to its high spatial and temporal resolution with nearly 100% sensitivity and specificity [26-28]. However, when involving the ascending aorta, type I and type II dissection imaging, even with 64 slices spiral CT and earlier DSCT, the pulsation artifact of the root-proximal ascending level cannot be eliminated [9,10]. Thus, the intimal tear location and intimal flap cannot be clearly displayed and cannot accurately measured. Some aortic type II dissections with limited intimal flap tear and close to the sinus are even misdiagnosed for an artifact because of limited intimal flap.

One recent study by Bolen et al. has shown that imaging of the thoracoabdominal aorta with ECG-triggered high-pitch CTA provides higher quality images of the aortic root and ascending aorta with sufficient contrast enhancement and decreased estimated radiation dose compared with non-ECG-synchronized standard-pitch helical CT [20]. However, the fast CTA scan speed of the whole aorta without the application of ECG-gating is especially important for the difficult patients, such as patients with urgent cardiovascular problems, restless patients and patients who cannot hold their breath for long. In this study, we compared the ascending aortic image quality and the whole aortic radiation dose of high-pitch DSCT angiography without ECG-gating and conventional DSCT angiography. The results of our study showed that the image quality of ascending aorta was much better with high-pitch mode than with the conventional scan mode, though the CTA image with pitch values up to 1.5 and without ECG is often degraded by pulsation artifacts at the aortic root. Recently, Beeres M et al. [29] reported that high-pitch CTA without ECG synchronization can provide motion-free aortic imaging, which our results totally agreed with. The motion-free imaging of the aorta without ECG synchronization may meet the clinical needs particularly for diagnosis. In addition, with high-pitch scanning mode and the application of the 100 kV low tube voltage, the patients in Group B received the effective radiation dose of only 2.7 ± 0.6 mSv, which was significantly lower than that of conventional protocol in Group A (3.9 ± 0.9 mSv).

Recent studies [17] showed that high-pitch mode can get coronary artery images with the diagnostic value, however, the heart rate had to be lower than 65 beats per minute and the heart rate variability had to be low. In our study, because of the low radiation dose and the high noise, only some openings of coronary arteries could be clearly displayed in the high-pitch mode group.

The main limitation of the present experiment was that a qualitative image quality scoring system was used in this study, which may be influenced by a subjectivity bias. On the other hand, our image quality kappa value of 0.87 corresponded to a good inter-observer agreement. Moreover, the effective dose calculated as the product of the DLP is based on phantom data and mathematic modeling and does not represent a patient-specific dose. However, effective dose determined in this way provides a reasonable estimate in clinical practice and aids in determining and comparing radiation risk for different CT protocols.

## Conclusions

Aortic dissection is the most frequently diagnosed lethal condition of the aorta. It is critical to make a prompt diagnosis of aortic dissection, which can decrease mortality and increase the survival rate. High-pitch scanning mode for DSCT can gain low radiation dose and excellent ascending aortic image quality at the same time in the whole aortic imaging, particularly in the aortic dissection imaging involving the ascending aorta.

## Abbreviations

CTA: CT angiography; DSCT: Dual-source CT; MPR: Multiplanar reconstruction; MIP: Maximum intensity projection; CPR: Curved planar reformation; VRT: Volume rendering technique; SNR: Signal to noise ratio; CTDIvol: Volume CT dose index; DLP: Dose length product; ED: Effective dose.

## Competing interests

One of the authors Hongtao Liu is Siemens employee.

## Authors’ contributions

YL and MWZ carried out the conception and design. JX and JL carried out the analysis and interpretation. JR, JQX, MQW and YWH participated in the data collection. YL and HTL participated in the writing the article, the critical revision of the article and the statistical analysis. All authors read and approved the final manuscript.
